# A semi-analytical method for characterization of fractal spoof surface plasmon polaritons with a transfer matrix and bloch theory

**DOI:** 10.1038/s41598-023-41050-3

**Published:** 2023-09-12

**Authors:** Vahid Najafy, Bijan Abbasi-Arand, Maryam Hesari-Shermeh

**Affiliations:** Department of Electrical and Computer Engineering, Tarbiat Modares University, Tehran, 14115-194 Iran

**Keywords:** Electrical and electronic engineering, Photonic devices

## Abstract

In this paper, a semi-analytical approach is introduced to analyze a spoof plasmonic structure, with an arbitrary geometry. This approach is based on a combination of techniques that employ a full-wave simulator and the Bloch theorem. By applying periodic boundary conditions, the real and imaginary parts of the equation obtained from the equivalent network have been calculated. To show the accuracy and validity of this proposed approach, a complementary Minkowski fractal SSPP unit cell has been designed and analyzed, and this has been used in a surface plasmonic transmission line. The results of our proposed method have been compared to measured results, and the simulated and measured results showed that the SSPP transmission line possesses high performance, from 1.45 to 5 GHz.

## Introduction

Surface plasmon polaritons (SPPs) are modes of surface waves, which are formed through the interaction between light waves, or any electromagnetic waves, and free electrons at the surface between a dielectric and a metal. As such, they are strongly dependent on the boundaries between the two environments^1^. The first synthetic SPP structures were introduced in 2004 by Pendry et al., under the name spoof SPPs. The method of creating this artificial structure was very similar to those of the artificial metamaterial structures that were created in 1996, by establishing negative permeabilities in these structures^2^.

Today, due to advances in technology, most wireless microwave systems, such as microwave imaging and sensor systems, have highly integrated and compact structures, with high qualities and small sizes. In these systems, signal accuracy, interference reduction, and device miniaturization have been the most important considerations and challenges; yet in most cases, these three parameters strongly conflict with each other. For example, by reducing the distance between a microstrip’s transmission lines, the interaction and coupling effects are increased, and this has a negative effect on the signal’s accuracy.

SSPP structures can be considered as suitable options, due to the similarity of their behavioral characteristics to SPPs—such as the lack of influence of any ambient waves on the carrier’s signals. Moreover, thanks to the strong dependence of spoof surface plasmon polaritons (SSPPs) on the boundaries between the two environments, the interaction effects between any two SSPP structures is also very low. This means that the protection and reliability of the signal in these structures is very high, and due to these features, devices based on these types of structures^3–6^ have smaller dimensions than conventional microwave devices^7^.

A dispersion curve is a powerful tool for expressing the main features of an SSPP structure. Although it is sometimes very difficult to achieve such diagrams for structures with arbitrary and complex shapes, it has not disappointed researchers to ignore this possibility^8^. Recently, engineers have proposed different methods for achieving dispersion curves. For example, in^9^, a circuit-model method based on the geometry of the structure was used to analyze the dispersion, and calculate the losses of the meander unit cell. In^8,10^, the combined field-network-joint method was employed, for the first time, to analyze the losses of SSPP structures, and also to analyze higher-order modes, where a periodic structure with bi-forked slits was investigated. Moreover, in^11^, a transfer matrix method was utilized to analyze both the scattering diagram and the transmission of plasmonic waves, in the conduction mode to the radiation mode.

Note that other conventional methods, such as mode matching or the modal method, have also often been used in the analysis of conventional structures, such as periodic solid cubes^12,13^, two-dimensional hole arrays^14^, periodic arrays of slanted grooves^15^, and gradient dielectric-filled metallic grating^16^. Furthermore, it is worth mentioning that commercial solvers (like CST eigenmode solver) are only able to calculate the frequency behaviors of the real parts of complex wave numbers, while the imaginary parts of the waves propagated in the SSPP structures cannot be displayed^17^.

Thus, there is a need for a more comprehensive method to be devised for obtaining the basic characteristics of these structures—such as the real and imaginary parts of the complex wave numbers of spoof plasmonic waves, and their impedance behaviors—regardless of the types and shapes of the structures. Recently, loss the analysis of reconfigurable spoof surface plasmon polaritons has also been done^18^. On the other hand, having a method that is independent of the shape of the unit cell and can also analyze reconfigurable SSPP structures is also useful. Since SSPP is one of the categories of one-dimensional periodic structures, it is useful to investigate different theories for analyzing one-dimensional periodic structures. Undoubtedly, having an analytical method for describing the properties of SSPP structures without any limitation in the shape of the unit cell and even reconfigurable SSPP structures will help the designer in implementing various microwave circuits and being active in this field^19,20^. are examples of combining SSPP structures with active chips that have led to the design and fabrication of active transmission lines (meta-channel).

Over the past few years, several methods have been presented for analyzing the dispersion of periodic structures. In^21^, in addition to calculating the dispersion relation for split-ring-resonator (SRR) unit cells, the authors also provided all the parameters required for systematic modeling, such as excitation and matching. In^22^, by presenting a 4-port circuit model and Bloch theory, the effects of coupling between the unit cells, for enhancing the bandwidth, was investigated and reported.

However, it is very complicated—if not impossible—to provide a circuit model for structures that have higher couplings and mutual effects, in higher-order modes. Therefore, in^17,23^, the dispersions of different structures including glide-symmetric holey EBGs and microstrip lines were analyzed through the combined methods of simulators. In those papers, the desired periodic structures were modeled as parallel and infinite combinations of equivalent unit cells, with each having a specific transfer matrix. Notwithstanding, the transfer matrix of a unit cell can be obtained by using full-wave simulators—from the values of the scattering matrices. Finally, the scattering analysis can be performed by applying the Bloch conditions to the boundaries of the unit cells.

In this paper, we analyze spoof plasmonic structures with arbitrary geometries, by using a combined method of implementing both simulation and Bloch theory^23^. To validate our method, a complex structure with a complementary Minkowski fractal SSPP (CMFSSPP) unit cell, which has been used in a surface plasmonic waveguide, is introduced and analyzed with our proposed approach. It is worth noting that this structure cannot be analyzed with other approaches presented in the literature ^8,9,12,24,25^. While using our proposed method, a detailed analysis of frequency behavior in higher frequency bands, and more analytical results than commercial solvers, such as loss and impedance, are presented. Moreover, a field-limited transmission line with the possibility of further miniaturization for various applications, including the integration of microwave circuits, is also provided using this new unit cell.

This paper is arranged as follows: In Sect. "Characterization of spoof surface plasmon polariton", the specification of a one-dimensional periodic structure is formulated, using the scattering matrix transformations and the transfer matrix of unit cells, as well as by applying the Bloch conditions. In Sect. "Design CMFSSPP unit cell", for validating the soundness of our proposed method in spoof plasmonic structures, a CMFSSPP unit cell is introduced, and the results are compared to the results of an Eigenmode solver for a CST simulation. A transmission line based on the CMFSSPP unit cell, with high-confinement capabilities, has been designed, and in Sect. "Transmission line based on CMFSSPP", the simulation results are compared to the results of a full-wave simulator. The measurement results are also presented to confirm the accuracy of our proposed method.

## Characterization of spoof surface plasmon polariton

**Figure 1:**
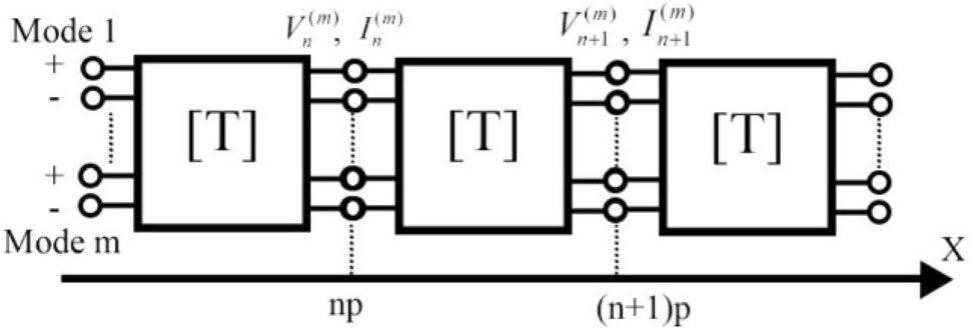
Equivalent network of the unit cell characterized by a multimodal transfer matrix.

Inspired by^23^, we assume that we have a one-dimensional periodic structure, with a period, p. The schematic view of each unit cell, which consists of a multipole with m inputs and outputs, is displayed in Fig. 1,

The symbols, ${{V}_{n}}^{(m)}$ and ${I}_{n}^{(m)}$, in the figure, represent the input voltage and input current of the *m*th mode; and ${V}_{n+1}^{(m)}$ and ${I}_{n+1}^{(m)}$ are the output voltage and current of the *m*th mode, respectively. This periodic structure can be modeled through a cascade connection of each of the multipoles, which have a separate transfer matrix, T (ABCD).

1 $$\left(\begin{array}{c}{V}_{n+1}\\ {I}_{n+1}\end{array}\right)=\left[T\right]\left(\begin{array}{c}{V}_{n}\\ {I}_{n}\end{array}\right)$$

In this case, we have the following:

2 $${V}_{n+1}=[{V}_{n+1}^{\left(1\right)},{V}_{n+1}^{\left(2\right)},...,{V}_{n+1}^{\left(m\right)}{]}^{T}$$

3 $${I}_{n+1}=[{I}_{n+1}^{\left(1\right)},{I}_{n+1}^{\left(2\right)},...,{I}_{n+1}^{\left(m\right)}{]}^{T}$$

4 $${V}_{n}=[{V}_{n}^{(1)},{V}_{n}^{(2)},...,{V}_{n}^{(m)}{]}^{T}$$

5 $${I}_{n}=[{I}_{n}^{(1)},{I}_{n}^{(2)},...,{I}_{n}^{(m)}{]}^{T}$$

The multimodal scattering parameters of the unit cell shown in Fig. 1 were simulated by CST frequency-domain solver, with hexahedral meshing and open boundaries set on the four physical ports at the side faces of the cell, each excited by *m* modes. The desired periodic structures were modeled as parallel and infinite combinations of equivalent unit cells, with each having a specific transfer matrix. Applying the Bloch condition to all the outputs leads to the following generalized eigenvalue problem, which is formulated through a general multimode transfer matrix:

6 $$\left(\begin{array}{c}{V}_{n+1}\\ {I}_{n+1}\end{array}\right)=\left(\begin{array}{c}{e}^{-i{K}_{x}P}{V}_{n}\\ {e}^{-i{K}_{x}P}{I}_{n}\end{array}\right)$$

In this equation, ${K}_{x}$ is the modal wave number (in general it is complex, and includes ${K}_{x}=\beta -i\alpha$), and ${e}^{-i{K}_{x}P}$ denotes the eigenvalue of the transfer matrix of each unit cell. For the general calculation of the transfer matrix, without losing the generality of the problem, it is sufficient to expose the desired structure as waves with different modes, in a full-wave simulator. Note that, in this article, due to the low amplitude of higher modes, only the dominant mode is considered, while its dispersion matrix is calculated for different frequencies, as follows.

The scattering matrix of this network can be written in terms of 4 partitioned m × m submatrices as:

7 $$\left[ S \right] = \left( {\begin{array}{*{20}c} {\left[ {S_{ii} } \right]} & {\left[ {S_{io} } \right]} \\ {\left[ {S_{oi} } \right]} & {\left[ {S_{oo} } \right]} \\ \end{array} } \right)$$

where the subscripts i/o represent the input–output ports.

8 $$\left[ T \right] = \left( {\begin{array}{*{20}c} {\left[ A \right]} & {\left[ B \right]} \\ {\left[ C \right]} & {\left[ D \right]} \\ \end{array} } \right)$$

Furthermore, using the following explicit relations, the transfer matrix can be easily calculated from the scattering matrix. In this case, I is the identity matrix, while Zi and Zo represent the characteristic impedance matrix of the input and output ports of the unit cell, respectively.

9 $$[\mathrm{A}]=\frac{1}{2}[([\mathrm{I}]+[{\mathrm{S}}_{\mathrm{ii}}])[{\mathrm{S}}_{\mathrm{oi}}{]}^{-1}([\mathrm{I}]-[{\mathrm{S}}_{\mathrm{oo}}])+[{\mathrm{S}}_{\mathrm{io}}]]$$

10 $$[\mathrm{B}]=\frac{1}{2}[([\mathrm{I}]+[{\mathrm{S}}_{\mathrm{ii}}])[{\mathrm{S}}_{\mathrm{oi}}{]}^{-1}([\mathrm{I}]+[{\mathrm{S}}_{\mathrm{oo}}])-[{\mathrm{S}}_{\mathrm{io}}]][{\mathrm{Z}}_{\mathrm{o}}]$$

11 $$[\mathrm{C}]=\frac{1}{2}[{\mathrm{Z}}_{\mathrm{i}}{]}^{-1}[([\mathrm{I}]-[{\mathrm{S}}_{\mathrm{ii}}])[{\mathrm{S}}_{\mathrm{oi}}{]}^{-1}([\mathrm{I}]-[{\mathrm{S}}_{\mathrm{oo}}])-[{\mathrm{S}}_{\mathrm{io}}]]$$

12 $$[\mathrm{D}]=\frac{1}{2}[{\mathrm{Z}}_{\mathrm{i}}{]}^{-1}[([\mathrm{I}]-[{\mathrm{S}}_{\mathrm{ii}}])[{\mathrm{S}}_{\mathrm{oi}}{]}^{-1}([\mathrm{I}]+[{\mathrm{S}}_{\mathrm{oo}}])+[{\mathrm{S}}_{\mathrm{io}}]][{\mathrm{Z}}_{\mathrm{o}}]$$

The variable, ${K}_{x}$, which satisfies Eq. (13), can be calculated at the Brillouin zone edge of the complex space of the propagated wave number, according to the following equation:

13 $$D({K}_{x},f)=\mathit{det}\{[T(f)]-[\Lambda ({K}_{x})]\}=0$$

In Eq. (13), $\Lambda$ is a matrix with $2m$ rows and columns, and the elements on the main diameter are equal to ${e}^{-i{K}_{x}P}$; and the other values of the elements are zero. In the end, through simple coding in MATLAB, it is possible to obtain the real and imaginary values of the calculated ${K}_{x}$ value, and to achieve the Bloch impedance value of the main mode of the structure, according to the following relationship, for different frequencies:

14 $${Z}_{in}={Z}_{0}\frac{[{V}_{n+1}]}{[{I}_{n+1}]}\overrightarrow{first mode}\frac{{Z}_{0}[B{]}_{1\times 1}}{\sqrt{([A{]}_{1\times 1}{)}^{2}-1}}$$

## Design CMFSSPP unit cell

Today, fractal structures are used in various applications, for different reasons such as in miniaturization and multiband devices^26^, boosting frequency bandwidths^27^, increasing isolation in array antennas^28^, and so on^29–31^. For this purpose, various fractal geometries have been presented, such as in Contour^32^, Minkowski^33^, and Koch^34^.

**Figure 2:**
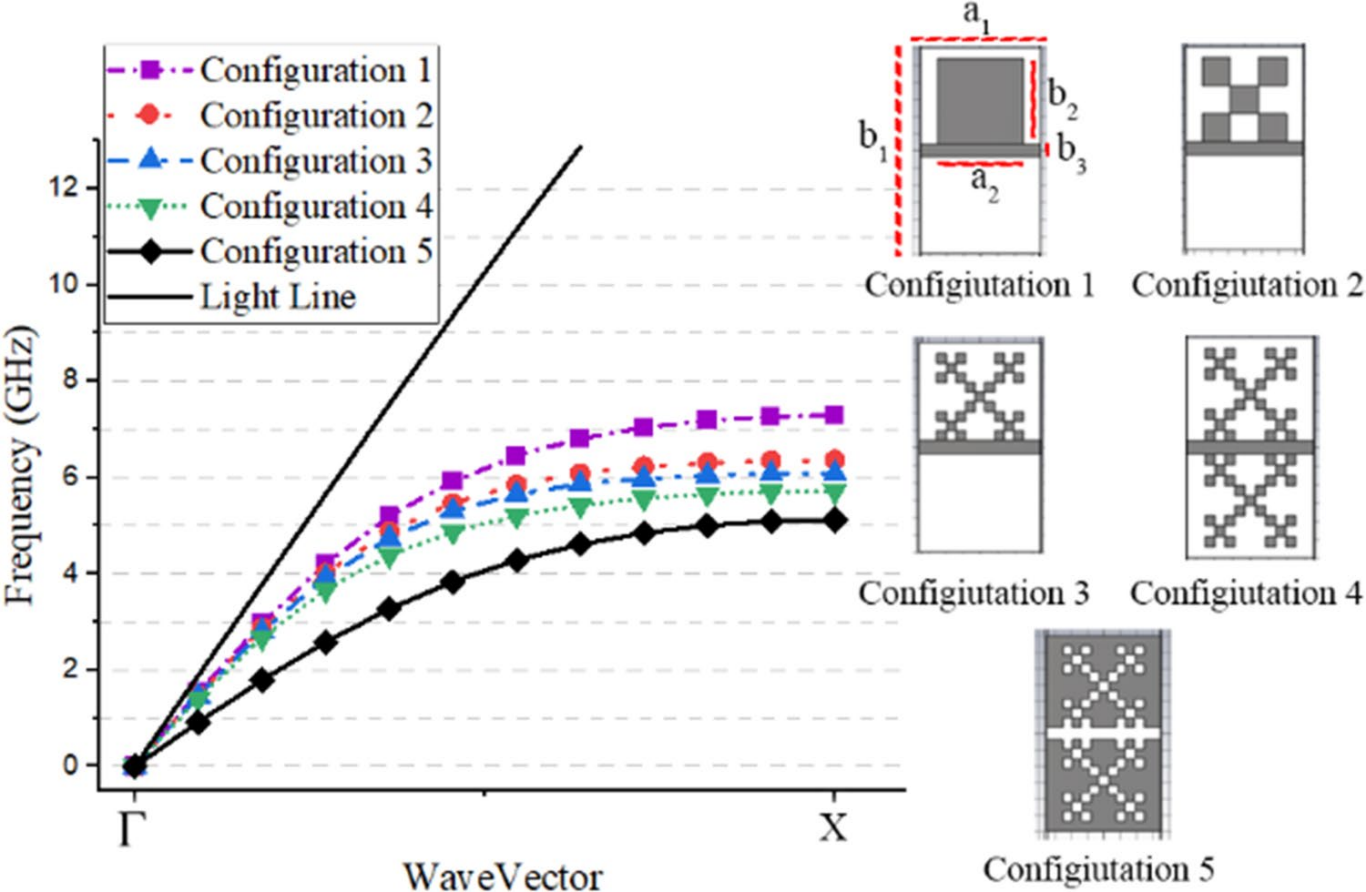
Comparison between the dispersion results for different steps of the unit cells’ design. The parameters are set as ${a}_{1}$, ${a}_{2}$, ${b}_{1}$, ${b}_{2}$, ${b}_{3}$, Scale 1, Scale 2 of 7, 5, 12, 5, 0.77, 0.3125 and 0.3125 mm respectively. The dielectric substrate, with a thickness of 1.6 mm, is FR-4, with a relative permittivity of 4.3, and a loss tangent of 0.025.

In order to validate the mentioned method^23^, in one-dimensional SSPP structures, a complementary Minkowski fractal SSPP (CMFSSPP) unit cell is presented, here. Since this unit cell has a complex structure, it is difficult to use the conventional method to provide an equivalent circuit for scattering analysis. Moreover, as can be seen, this structure has a greater field confinement, and a lower wave propagation speed than similar SSPPs^35^; where the dispersion results of the proposed unit cell are presented in Fig. 2.

**Figure 3:**
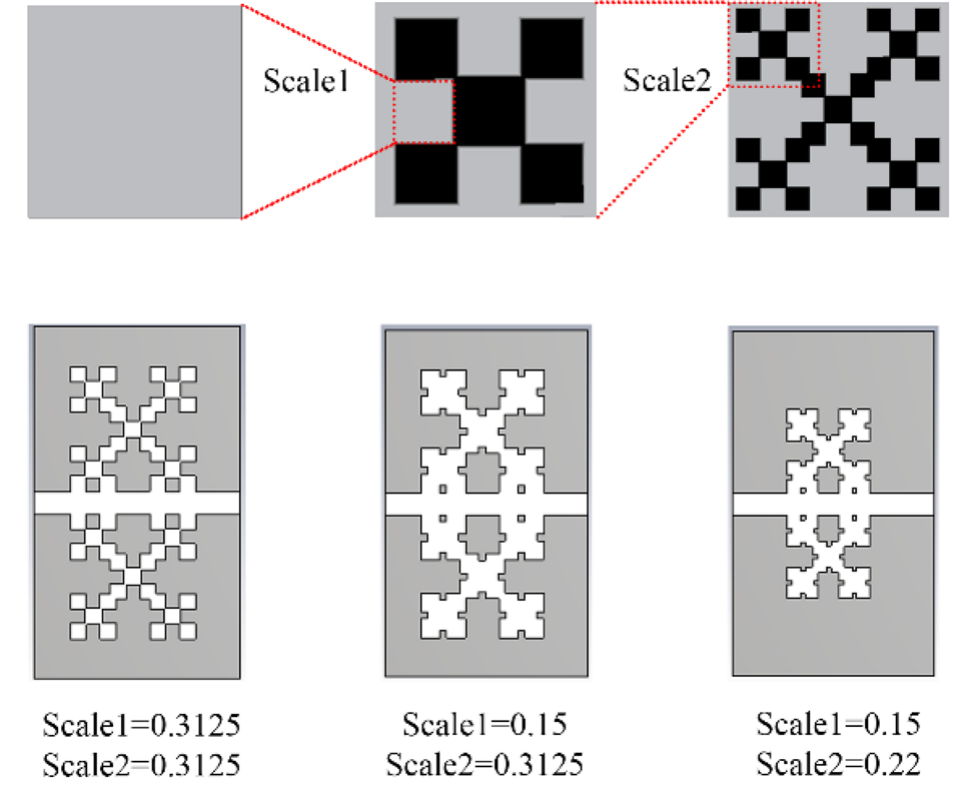
The Scale 1 and Scale 2 parameters of the three unit-cells.

**Figure 4:**
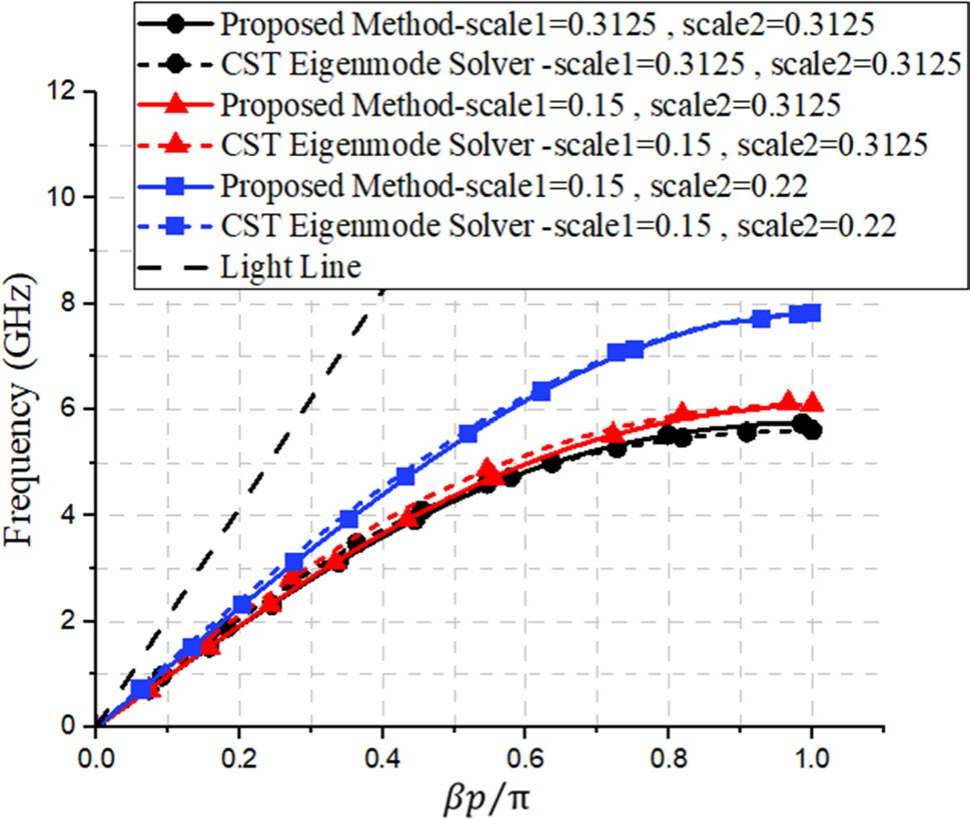
The dispersion results of the SSPP modes for the three unit-cells presented in Fig. 3. The results of our proposed method are shown in comparison, here, to the results of a CST Eigenmode solver.

**Figure 5:**
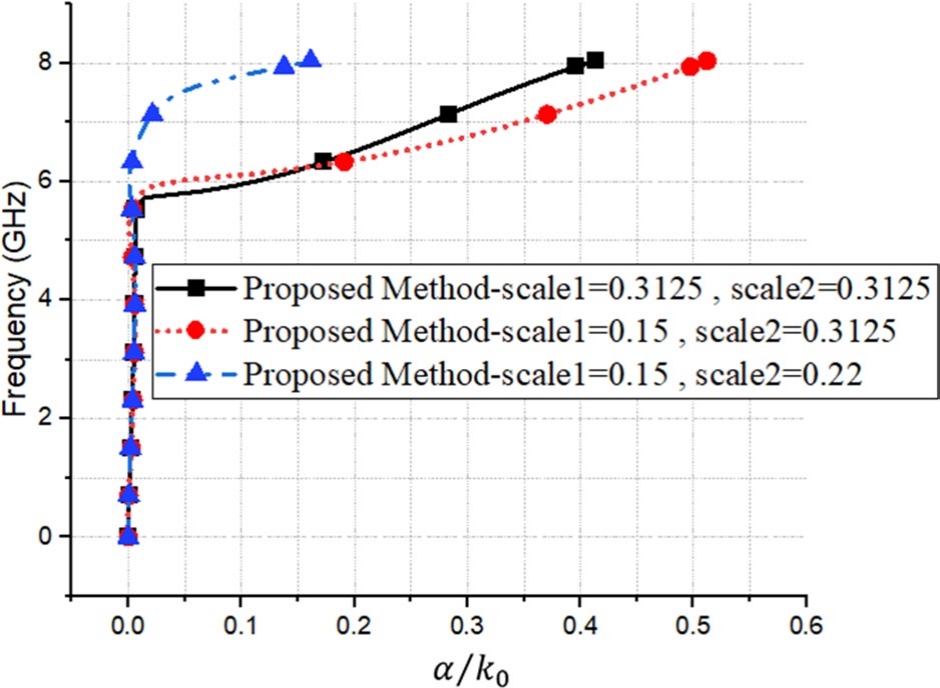
The effects of different values of Scale 1 and Scale 2, on the attenuation results.

**Figure 6:**
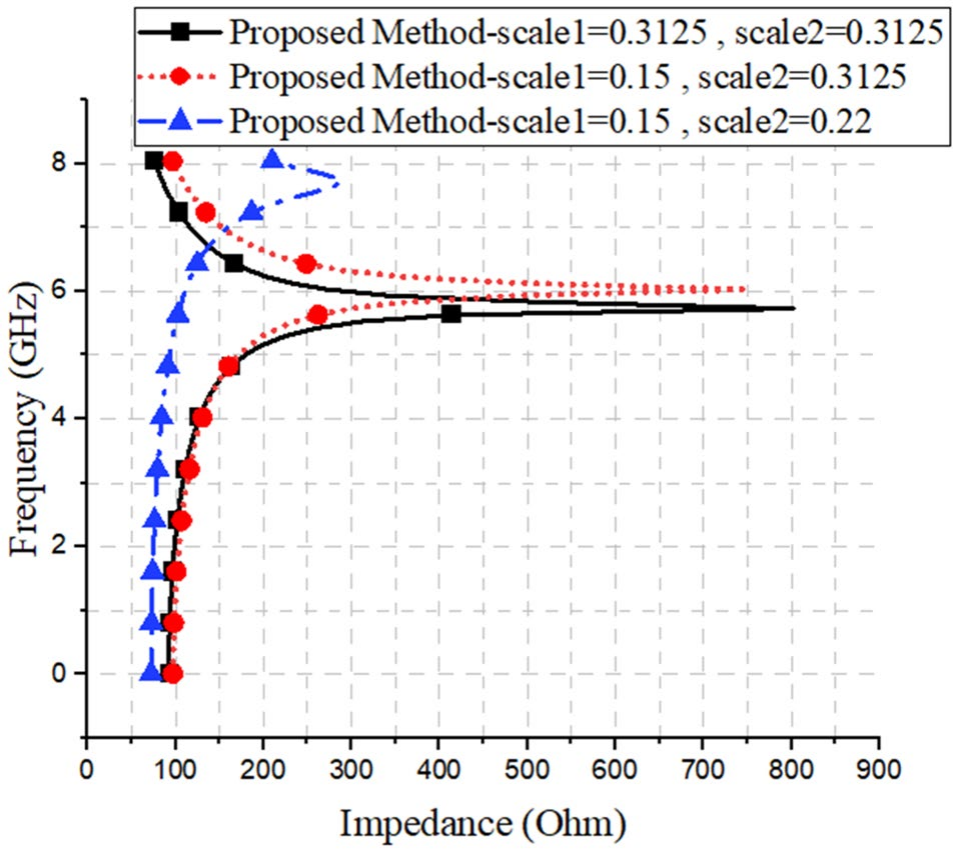
The effects of different values of Scale 1 and Scale 2, on the impedance results.

As can be seen in the figure, the proposed structure is presented separately, and the final structure has been obtained in the fifth stage. It consists of a slot line and two complementary Minkowski fractal structures, which can be managed by the two parameters, Scale 1 and Scale 2 (see Fig. 3 for more clarification). In Fig. 2, by comparing the results of the configurations 1 and 5, at the same plasma frequencies, there is a 31% size reduction of the proposed unit cell, compared to a conventional unit cell. Moreover, the results of the dispersion, loss, and impedance of three unit-cells with the values specified in Fig. 3, are illustrated in Figs. 4, 5 and 6, respectively.

As mentioned previously, it is worth noting that, in contrast to our proposed approach, commercial software can only calculate the dispersion diagram of a unit cell (as shown in Fig. 4), and it cannot calculate the other characterization values, such as the loss or impedance behaviors of the SPP structures (as shown in Figs. 5 and6). However, it should be noted that for some specific unit cells, transmission line-based methods have been proposed to investigate the impedance behavior of SSPP structures^36^.

## Transmission line based on CMFSSPP

**Figure 7:**
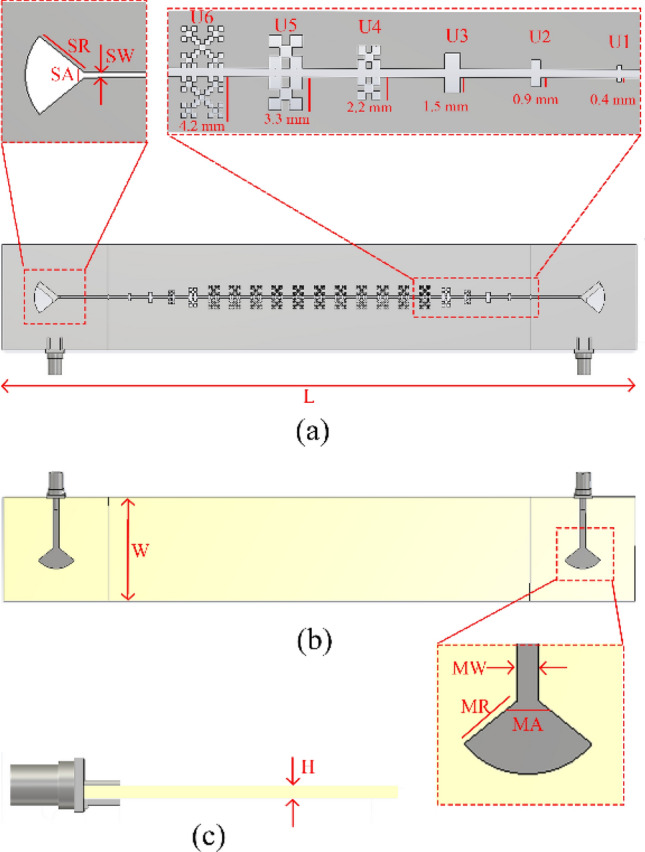
(**a**) Front view, (**b**) rear view, and (**c**) side view of the transmission line. The SR, SA, SW, L, W, MR, MA, and MW parameters were set as 9 mm, 78°, 0.8 mm, 240 mm, 40 mm, 7.37 mm, 101°, and 2.31 mm, respectively.

In this section, to validate the results of the proposed unit cell, as well as the application of the results extracted from our presented method, in the design of microwave devices based on SSPP, a transmission line based on SSPP has been designed and fabricated. This transmission line was designed consisting of two microstrip-to-slot-line transitions, and these were designed by a slot line on which the SSPP unit cells were placed with a period of 8 mm. The schematic of the structure is shown in Fig. 7.

**Figure 8:**
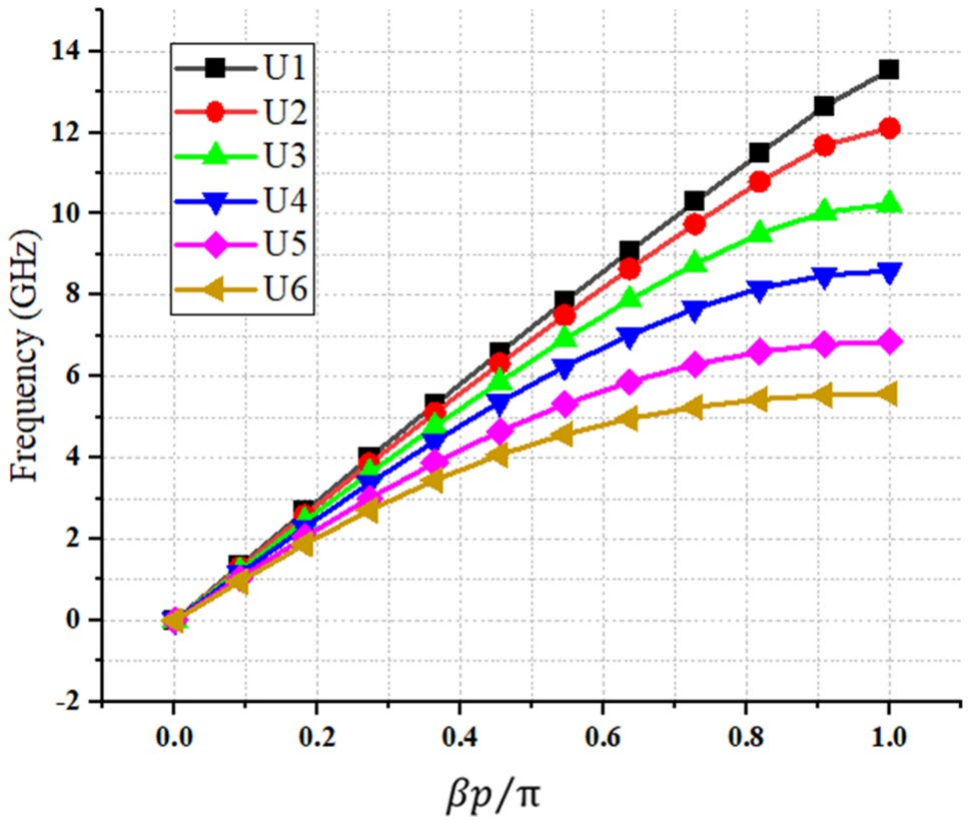
Dispersion characteristics of the transition structure for different iterations. The parameters are shown in Fig. 7a.

As shown in Fig. 6, the impedance of the unit cells, at different scales, could be considered with an average of around 120 ohms, and within a frequency range of 1.5–5 GHz. Bearing this in mind, while designing the transmission line, it is tried to use two 50-Ω coaxial line transitions to 120-Ω slot lines. These transitions consisted of a microstrip line on the substrate, and a ground plane with a slot line of a certain width, on the other side. These two lines were also perpendicular to each other, and both the microstrip and slot lines had radial stubs that helped to increase the bandwidths of the impedance matching. Moreover, 20 SSPP unit cells, with periods of 8 mm, were placed in the spaces between the two transitions, to achieve maximum impedance and momentum matching. The unit cells were also arranged in descending orders of plasma frequencies, and their detailed view and dispersion results are shown in Figs. 7a and 8, respectively. The substrate used was conventional FR4, with a thickness of 1.6 mm, and a loss tangent of 0.025; and the other parameters and sizes are presented in the caption of Fig. 7.

**Figure 9:**
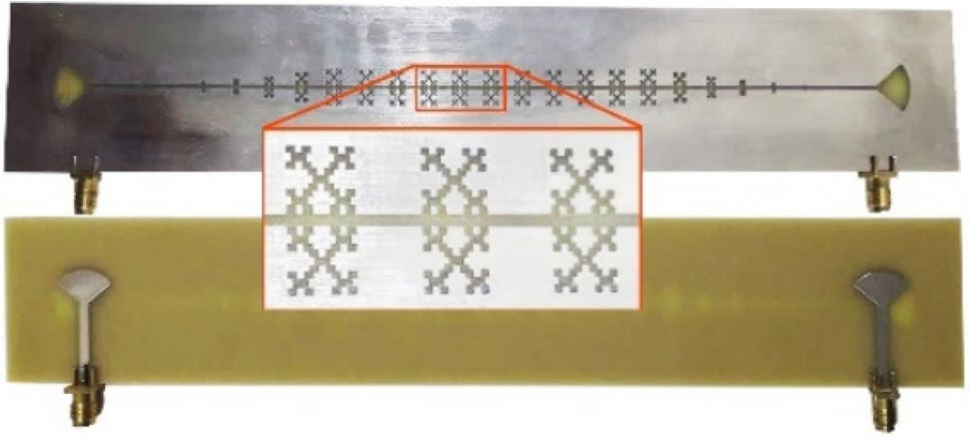
The fabricated SSPP transmission line.

**Figure 10:**
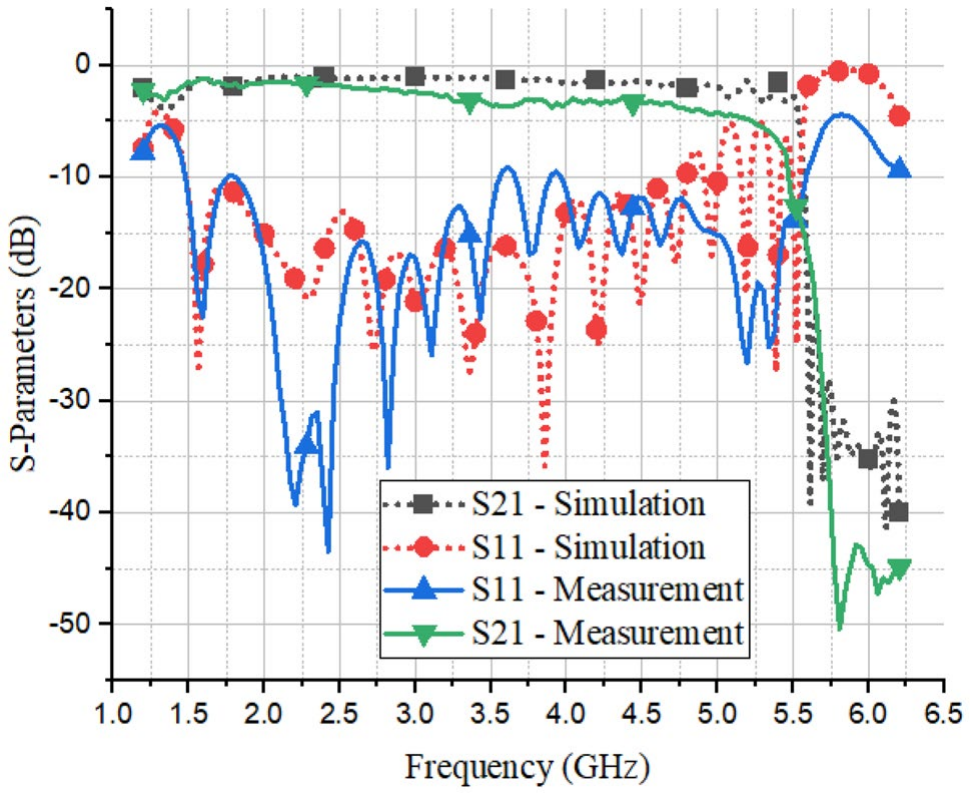
A comparison of the measurement and simulated S-parameters of the proposed SSPP transmission line.

Next, in order to validate our proposed analysis method, as well as present a transmission line based on CMFSSPP, the corresponding structure was fabricated, as shown in Fig. 9. Figure 10 shows the measurement results that were obtained with a network analyzer, and these were in good agreement with our simulation results. Table 1 also shows a comparison of our presented analysis method, and the mentioned references. The comparison of the new unit cell, as presented with the conventional example depicted in Fig. 2, together with its associated dispersion diagram and plasma frequency, indicated that there was a 31% reduction in the dimensions of the presented unit cell, and this has significant implications for their use in the miniaturization of microwave devices.

**Table 1:** A comparison of the proposed characterization method, in relation to previous studies.

Reference	^25^	^9^	^8^	This work
Dependence on the shape of the structure	Have	Have	Have	Do not have
Accuracy	High	Medium	High	High
Complexity	Medium	Low	Medium	Low
Ability to analyze higher modes	Have	Do not have	Have	Have

## Conclusion

In this paper, a semi-analytical method for the characterization of fractal spoof surface plasmon polaritons, with a transfer matrix and Bloch theory, has been presented. This method can be used for arbitrary periodic structures, such as complex SSPPs, yet it provides more analytical results than commercial solvers like CST eigenmode solver. In order to validate our presented method, and assess the performance of the new unit cell, a transmission line based on this idea has been designed and fabricated, and a comparison of the simulation and measurement results has verified its impressive performance within the frequency range of 1.45–5 GHz.

## Data Availability

The datasets generated and analyzed during the current study are available from the corresponding author on reasonable request.
